# Fifty years of limnological data on Lake Stechlin, a temperate clearwater lake

**DOI:** 10.1038/s41597-025-05319-8

**Published:** 2025-06-18

**Authors:** Sabine Wollrab, Silke R. Schmidt, Jason Woodhouse, Peter Kasprzak, Stella A. Berger, Ute Beyer, Matthias Bodenlos, Johanna Dalchow, Monika Degebrodt, Lars Ganzert, Thomas Gonsiorczyk, Elfi Huth, Christine Kiel, Lutz Küchler, Lothar Krienitz, Maren Lentz, Elke Mach, Uta Mallok, Jens C. Nejstgaard, Monika Papke, Armin Penske, Solvig Pinnow, Reingard Roßberg, Diethelm Ronneberger, Michael Sachtleben, Adelheid Scheffler, Hans-Peter Grossart, Peter Casper, Mark O. Gessner, Rainer Koschel

**Affiliations:** 1 Leibniz Institute of Freshwater Ecology and Inland Fisheries (IGB), Department of Plankton and Microbial Ecology, Stechlin, Germany; 2 German Environment Agency (UBA), Application Lab for AI and Big Data, Buchholzweg 8, 13627 Berlin, Germany; 3 Universität Hamburg, Institute of Cell and Systems Biology of Animals, Martin-Luther-King Platz 3, 20146 Hamburg, Germany; 4 University of Potsdam, Institute of Biochemistry and Biology, Maulbeerallee 2, 14469 Potsdam, Germany; 5 Berlin Institute of Technology (TU Berlin), Department of Ecology, Ernst-Reuter-Platz 1, 10587 Berlin, Germany

**Keywords:** Limnology, Biogeochemistry, Freshwater ecology, Climate-change ecology

## Abstract

We present 50 years of monitoring data on water quality of Lake Stechlin, a deep, dimictic hardwater lake in northeastern Germany known for its exceptionally clear water. Although located in a forested catchment, the lake has undergone major changes in recent decades, including a period of massive heating of surface water when receiving cooling water from a nearby nuclear power plant (1966–1990), accompanied by a greatly shortened water residence time from more than 40 years to less than 300 days. These changes are superimposed by a long-term trend of surface water warming and a concomitant decrease in winter ice cover. Total phosphorus concentrations have quadrupled since 2010 and zones of deep-water oxygen depletion have greatly expanded. The presented dataset covers basic water-chemical and physical records taken at monthly to fortnightly intervals from 1970 to 2020, documenting limnological changes during that period. Furthermore, it serves as a valuable basis to assess and project potential consequences of climate change and other types of environmental change on deep clearwater lakes in temperate climates.

## Background & Summary

Although covering less than 1% of the earth surface, lakes and other freshwater ecosystems are hotspots of biodiversity, contribute significantly to global biogeochemical cycles, and provide important ecosystem services such as drinking water supply, inland fisheries and recreation^1–3^. At the same time, lakes are vulnerable to impacts of climate change through a range of mechanisms^4^. These include globally rising temperatures^5–7^, changes in precipitation regimes^8^, and increases in the intensity and frequency of extreme weather events such as severe storms^9–11^. Warming directly affects lake stratification, mixing and oxygen regimes with consequences for biogeochemical processes^7,12^. Similarly, changes in precipitation regimes and storms influence carbon and nutrient supplies from the catchment in addition to affecting the physical conditions of lakes^13,14^. Long-term data records have been instrumental in documenting such impacts^5,15,16^ and are an important asset to assess and project future consequences of ongoing climate change on physical^17,18^, biogeochemical^19^ and biological variables^20–23^. In addition to documenting change, these data, together with advances in modelling and statistics, now also facilitate the attribution of causes to climate change and other factors in multi-stressor contexts^24–26^. Here we present five decades of curated physical and water-chemical data on a deep clearwater lake in Central Europe, Lake Stechlin, which has been exposed to multiple stressors and has undergone important changes over the reported monitoring period.

**Fig. 1:**
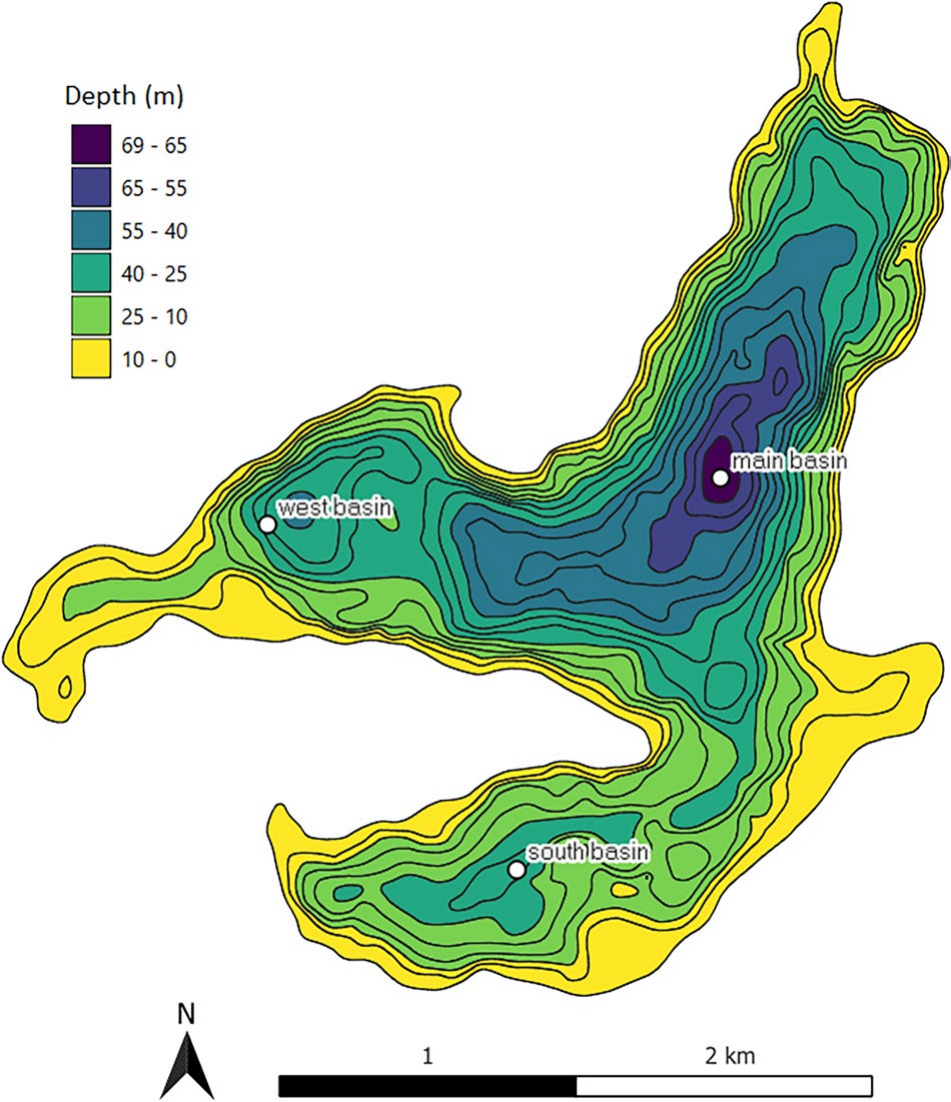
Bathymetric map of Lake Stechlin with three sampling points used during the monitoring programme and referred to as main, west and south basin.

Lake Stechlin is a clear hardwater lake located in the lake district of northeastern Germany (53°9′5.6″N, 13°1′34.2″E). The lake is situated at 59 m altitude in an 86.6 km^2^ nature conservation area south of the Baltic Sea and 80 km north of the city of Berlin. The maximum depth is 69.5 m at a mean depth of 23.3 m, a surface area of 4.23 km^2^ and a volume of 98.7 × 106 m^3^ ^27^ (Fig. 1). The three basins of the lake were formed during the last continental glaciation ca. 12,000 years ago. Today, the area is situated at the transition between temperate maritime and temperate continental climate^28^. The small catchment (12.6 km^2^, including the lake surface) is dominated by sandy soils overlying till of a endmoraine, and almost completely covered by managed forest (95%) with Scots pine (*Pinus sylvestris*) as the main species, although beech (*Fagus sylvatica*) dominates the tree vegetation along the shoreline^29^. Non-forested areas in the catchment are limited to the premises of a former nuclear power plant and to a small village (Neuglobsow) with about 300 residents and up to 700 visitors during the summer tourist season. Wastewater from the village is diverted to a treatment plant outside of the catchment. The indented shoreline is largely undeveloped with no notable infrastructure, except one small beach and a professional fisherman, a small boat rental, a field station of the German Environment Agency, and the Leibniz Institute of Freshwater Ecology and Inland Fisheries (IGB). Originally endorheic, the lake is primarily fed by direct precipitation on the water surface and by ground water, resulting in a theoretical water residence time exceeding 40 years^30,31^. There is no inflow, except for occasional discharge from a small channel that is dry in most years. Water losses mainly occur by evaporation and as groundwater outflow.

A canal connecting Lake Stechlin to the River Havel via neighbouring Lake Nehmitz was built between 1746 and 1750, lowering the water level by about 1 m. The canal between Lakes Stechlin and Nehmitz was re-activated in 1960. A weir has since regulated discharge and thus the water level of the upstream lakes, including Lake Stechlin^27,29,32^. From 1966 to 1990, Lake Stechlin received a total of about 300,000 m^3^ d^−1^ of cooling water from a nearby nuclear power plant (70 MW nominal capacity). The cooling water was withdrawn from the north basin of Lake Nehmitz and discharged into Lake Stechlin at an average temperature of approximately 10 °C above the ambient surface water temperature^29,33,34^. This increased the lake’s annual average surface water temperature by 1–2 °C during the power plant operation and shortened the water residence time to less than 300 days^29,33,34^. Further information on the lake, its catchment and its history can be found in the following publications^27,29,34,35^.

**Fig. 2:**
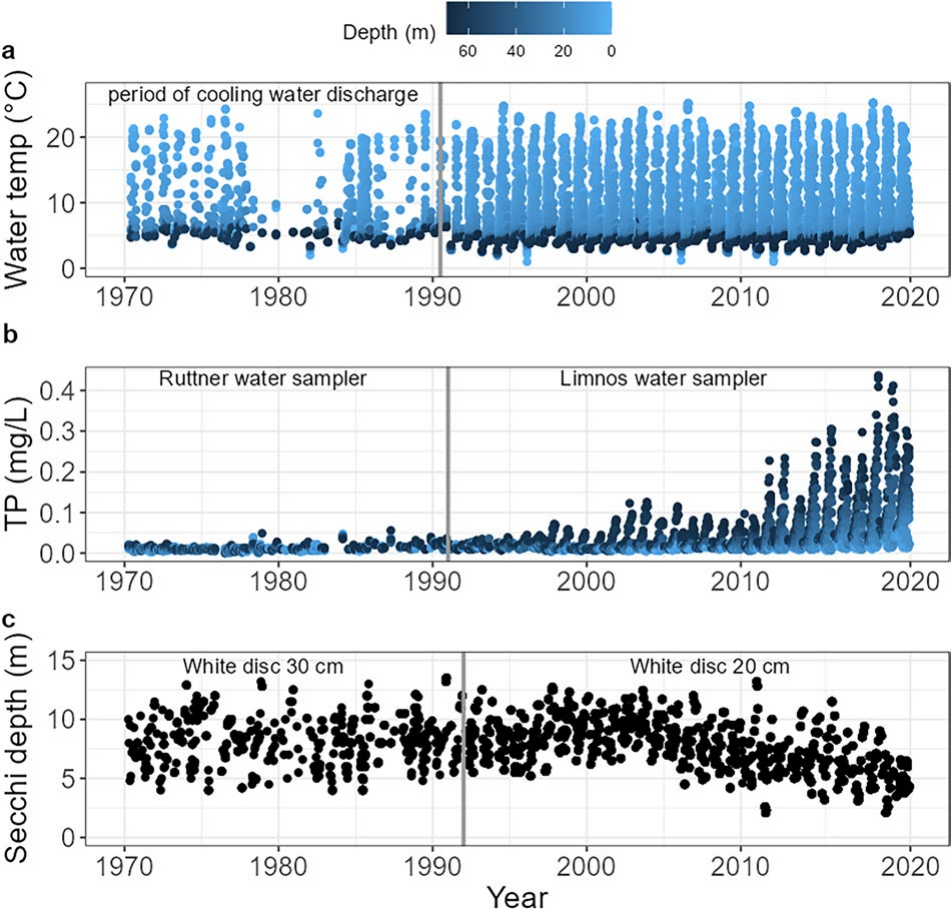
Time series of (**a**) water temperature, (**b**) total phosphorus concentration (TP), and (**c**) water transparency (Secchi depth) determined in the main basin of Lake Stechlin between 1970 and 2020. In panels a and b, symbols of sampling values are coloured according to sampling depth, ranging from surface measurements (0 m) in light blue to 65 m below surface in black. Grey vertical lines indicate (**a**) end of period of cooling water discharge, (**b**) change in water sampler from Ruttner to Limnos, as well as (**c**) change from Secchi depth measurements using a 30 cm to a 20 cm white disc.

Lake Stechlin has traditionally been known for its clear water^27,29^, as also implied by its name of Slavic origin meaning glass. In recent years, however, the lake has undergone major changes. Winter ice cover has diminished, and despite important interannual variation, an increasing trend of surface water temperatures is clearly apparent, independent of the warming effect caused by the power plant^33,36^ (Fig. 2a). The mixing regime is gradually shifting from dimictic to monomictic^12^. Additionally, precipitation is declining in the region, especially during summer^37^, and the frequency of severe storms has increased^38,39^. A major storm that hit Lake Stechlin in the summer of 2011 disrupted the vertical thermal structure and entrained a dense layer of cyanobacteria into the sunlit epilimnion^9^. This triggered a pronounced algal bloom that persisted for weeks and led to massive calcite precipitation and a dramatically reduced water transparency. Even more striking, Lake Stechlin has experienced a quadrupling of total phosphorus concentrations since 2010^19^ (Fig. 2b), accompanied by an increase in phytoplankton abundance^40,41^, reduced water transparency (Fig. 2c), and expanding deep-water oxygen depletion leading to extensive anoxic deep-water zones during summer stratification^41–43^.

A limnological monitoring programme on Lake Stechlin was started in the 1960s concomitant with the construction of the nuclear power plant on the lake shore. The Research Department for Limnology as part of the network of the Academy of Sciences of the German Democratic Republic (GDR) and the Research Institute of Hydrometeorology of the Meteorological Service of the GDR (after 1992 part of the German Weather Service) led the monitoring and research programme on Lake Stechlin and its catchment^29,44–46^. The monitoring programme was expanded in the 1970s, but sampling was irregular before 1990, which resulted in notable data gaps during the 1980s. The most complete data set is available for the period since 1992, when the laboratory became a department of the newly founded IGB. Here we present water-chemical and physical data on Lake Stechlin for the period from 1970 to 2020 (time series for selected parameters illustrated in Fig. 2). A complete list of parameters, analytical methods and sampling periods is presented in Table 1. Long-term hydrometeorological data have also been collected and are partly published^46^. Data on the composition, and abundance of plankton and pelagic fish are compiled in separate data packages^40,41,47^.

**Table 1:** List of the measured parameters, methods used and corresponding time spans.

Parameter	Abbreviation	Unit	Method	Time span
Secchi depth	secchi	m	Secchi disk	1970–2020
Water temperature	T	°C	probe	1970–2020
Dissolved oxygen	O_2_	mg L^−1^	titrimetry (Winkler) probe	1970–1991
			probe	1992–2020
Oxygen saturation	%O_2_	%	titrimetry (Winkler)	1970–1991
			probe	1992–2020
pH	pH		probe	1971–2020
Specific conductivity	conductivity	µS cm^−1^	probe	1990–2020
Turbidity	Turbidity	NTU	probe	2013–2020
Chlorophyll *a*	chl *a*	µg L^−1^	probe	2013–2020
Phycocyanin	PC	cells L^−1^	probe	2013–2020
Soluble reactive phosphorus	SRP	mg L^−1^	spectrophotometry	1970–2020
Total phosphorus	TP	mg L^−1^	spectrophotometry	1970–2020
Ammonium	NH_4_^+^	mg L^−1^	spectrophotometry	1970–2020
Nitrite	NO_2_^−^	mg L^−1^	spectrophotometry	1970–2020
Nitrate	NO_3_^−^	mg L^−1^	spectrophotometry	1970–2020
Dissolved silicon	Si	mg L^−1^	spectrophotometry	1971–2020
Chlorophyll *a*	chl *a*	µg L^−1^	spectrophotometry	1980–2020
Total alkalinity	Alkalinity	mmol L^−1^	titrimetry	1984–2020
Calcium carbonate	CaCO_3_	mg L^−1^	Infrared gas analyser	1988–2020
Total nitrogen	TN	mg L^−1^	spectrophotometry	1993–2020
Dissolved calcium	Ca^2+^	mg L^−1^	spectrophotometry	1993–2020
Non-purgeable organic carbon	NPOC	mg L^−1^	catalytic oxidation, NDIR	1994–2020
Total iron (Fe^2+^ + Fe^3+^)	Fe	mg L^−1^	spectrophotometry	1996–2020
Total inorganic carbon	TIC	mg L^−1^	NDIR	1996–2020
Dissolved aluminium	Al	mg L^−1^	spectrophotometry	2003–2020
Dissolved organic carbon	DOC	mg L^−1^	catalytic oxidation, NDIR	2013, 2015
Sodium	Na^+^	mg L^−1^	Ion chromatography	2000–2020
Magnesium	Mg^2+^	mg L^−1^	Ion chromatography	2000–2020
Calcium	Ca^2+^	mg L^−1^	Ion chromatography	2000–2020
Chloride	Cl^−^	mg L^−1^	Ion chromatography	2000–2020
Sulphate	SO_4_^2^^−^	mg L^−1^	Ion chromatography	2000–2020
Potassium	K^+^	mg L^−1^	Ion chromatography	2007–2020

## Methods

### Sampling

Water samples for chemical analyses have been taken at the deepest point of the lake (69.5 m) in the main basin (53°9′19.5″N, 13°1′52.9″E) since 1970. Additional samples were collected in the west (53°9′15.1″N, 13°0′30.5″E) and south (53°8′37.0″N, 13°1′14.9″E) basin from 1985 onwards (Fig. 1), complemented between 1994 and 2018 by samples from the eastern bay, which occasionally received surface water inflow. Sampling frequency varied over the years. In the main basin, fortnightly samples were usually taken from May to September, although various measurements were only made monthly. Monthly samples were also taken during the rest of the year, unless winter ice conditions prevented access to the main sampling site either by boat or on foot. In the south and west basin, sampling was restricted to the end of summer stratification in either November or December. However, both of these basins were sampled seven times in 2020.

Water samples from the surface mixed layer were taken using a 1-L or 2-L Ruttner sampler between 1970 and 1990. A 5-L water sampler (Hydro-Bios, Kiel, Germany) was occasionally used from 1985 onwards, but the sampler routinely used since 1991 has been a 2.6-L or 3.5-L Limnos water sampler (Limnos Oy, Turku, Finland). Before 1992, epilimnion (surface mixed layer) samples were taken at 2.5 m intervals from 0 to 10 m depth. These samples were analyzed separately. Since 1992, epilimnetic samples have been taken at 0 and 5 m depth and pooled, with a sample taken at 10 m depth added when the epilimnion was deeper. A pooled sample from 0 and 2.5 m depth was occasionally used during periods when the epilimnion was very shallow. Hypolimnetic water was collected at 15, 20, 40, 60 and 65 m depth before 1992, and at 40, 60 and 65 m depth between 1992 and 2008. These discrete hypolimnetic samples were separately analyzed. Since 2009, additional discrete samples have been taken during summer stratification at 5 m intervals between 15 and 55 m depth.

### Field measurements

Water transparency was determined as Secchi depth (secchi) on each sampling occasion. A white disc 30 cm (1970–1991) or 20 cm (1992–2020) in diameter was lowered in the water column until it was no longer visible, then raised, and the depth recorded both when the disc disappeared and when it re-appeared. The mean of both values is reported as Secchi depth. Readings were taken with a bathyscope on the shady side of a boat to reduce the influence of reflections and glittering. Before 1992, water temperature was measured using the mercury thermometer of the Ruttner sampler^48^ and dissolved oxygen concentrations were determined by the Winkler method^48,49^. From 1992 onwards, multi-parameter probes were used to obtain vertical profiles (1–5 m depth intervals) of temperature, dissolved oxygen, oxygen saturation, pH, specific conductivity, and, from 2013 onwards, turbidity, chlorophyll *a* (chl *a*) and phycocyanin (PC). Hand-held WTW multiprobes (OXI-197, Weilheim, Germany) were used until 2009, and YSI multiprobes (YSI 6600, Yellow Springs, OH, USA) since 2010. Sensors were regularly calibrated in the lab according to the user manuals.

### Water-chemical analyses

Concentrations of total phosphorus (TP), soluble reactive phosphorus (SRP), total nitrogen (TN), ammonium (NH_4_^+^), nitrite (NO_2_^−^), nitrate (NO_3_^−^), dissolved calcium (Ca^2+^), dissolved aluminium (Al), dissolved iron (Fe: sum of Fe^2+^ and Fe^3+^) and dissolved silicon (Si), total inorganic carbon (TIC), non-purgeable organic carbon (NPOC) (i.e. the fraction of organic carbon remaining after sparging an acidified aqueous sample with gas to remove all volatile carbon constituents), dissolved organic carbon (DOC), calcium carbonate (CaCO_3_), total alkalinity (alkalinity) and chl *a* were determined following standard methods or protocols outlined in the user manual of the respective instrument (see below for details). Concentrations of dissolved anions chloride (Cl^−^) and sulphate (SO_4_^2−^) as well as cations sodium (Na^+^), potassium (K^+^), magnesium (Mg^2+^) and calcium (Ca^2+^) were determined by ion chromatography with conductivity detection. All analyses of dissolved components (SRP, NO_2_^−^, NO_3_^−^, NH_4_^+^, Ca, Al, Fe, Si, DOC, and all other anions and cations) were performed on water samples passed through NC60 membrane filters (0.6 µm pore size, Whatman, Little Chalfont, United Kingdom) before July 2010, cellulose nitrate membrane filters (0.45 µm pore size, Whatman, Little Chalfont, United Kingdom) between July 2010 and April 2012, and cellulose acetate membrane filters (0.45 µm pore size, Sartorius AG, Göttingen, Germany) since May 2012. Unless analysed immediately after filtration, samples were stored at −20 °C. The residual filters were used for CaCO_3_ determination, were dried at 60 °C overnight and stored in a desiccator until analysis. Filters for chl-*a* analyses were stored at −20 °C. TP and TN were measured after wet digestion of unfiltered aliquots in an autoclave using 50 g L^−1^ potassium peroxodisulfate as oxidant for TP (30 min at 134 °C) and oxidising decomposition reagent (Oxisolv®,Merck, Germany) for TN (45 min at 120 °C).

Details of the analytical methods are as follows:

- TP, SRP: Spectrophotometry
  -   Before 1992, manual spectrophotometric analysis^48,50–53^
  -   From 1992 to December 2005, flow-injection analysis with spectrophotometric detection: FIAstar (Tecator AB, Höganäs, Sweden). Application Note 60/83, ASN 60-03/83 (Determination of orthophosphate in water by flow injection analysis) and ASN 60-04/83 (Determination of total phosphate in water by flow injection analysis)
  -   From January 2006 to December 2014, flow-injection analysis with spectrophotometric detection: FIAcompact (Medizin- und Labortechnik GmbH, Dresden, Germany). Arbeitsanleitung Orthophosphat in Wasser/ Abwasser/ Bodenextrakten, fotometrisch über Phosphomolybdat und Reduktion zu Molybdänblau, 2005, based on DIN EN ISO 15681-1
  -   Since January 2015, flow-injection analysis with spectrophotometric detection: FIAstar 5000 Analyzer (Foss Analytical AB, Höganäs, Sweden). Application Notes 5240 (Determination of ortho-phosphate in water by FIAstar 5000) and 5241 (Determination of total phosphorus in water by FIAstar 5000) according to ISO 15681-1

- TN, NO_2_^−^, NO_3_^−^, NH_4_^+^: Spectrophotometry
  -   Before 1992, flow stream analysis^54–57^, conducted by Wasserwirtschaftsdirektion Havel, Potsdam, German Democratic Republic^48^
  -   From 1992 until October 2009, flow-injection analysis with spectrophotometric detection: FIAstar (Tecator AB, Höganäs, Sweden). Application Note 62/83, ASN 110-01/92 (Determination of the sum of nitrate and nitrite in water by flow injection analysis) and ASN 110-03/92 (Determination of total nitrogen in water by flow injection analysis), and Application Note 50/84, ASN 151-01/92 (Determination of ammonia nitrogen in water by flow injection analysis) according to DIN 38406 (E23)
  -   Since November 2009 flow-injection analysis with spectrophotometric detection: FIAstar 5000 Analyzer (Foss Analytical AB, Höganäs, Sweden). Application Note 5201 (Determination of the sum of nitrate and nitrite in water by FIAstar 5000) according to ISO 13395-1996, Application Note 5202 (Determination of total oxidized nitrogen in water by FIAstar 5000) according to ISO 11905 and ISO 13395, and Application Note 550 (Determination of ammonium in water by FIAstar 5000) according to ISO 11732

- Ca^2+^: Spectrophotometry,
  -   From 1992 until October 2009, flow-injection analysis with spectrophotometric detection: FIAstar (Tecator AB, Höganäs, Sweden). Application Note 48/83, ASN 48-03/84 (Determination of calcium in water by flow injection analysis)
  -   Since November 2009, flow-injection analysis with spectrophotometric detection: FIAstar 5000 Analyzer (Foss Analytical AB, Höganäs, Sweden). Application Note 5261 (Determination of dissolved calcium in water by FIAstar 5000) according to DIN 38406-3

- Al: Spectrophotometry, flow-injection analysis with spectrophotometric detection: FIAcompact (Medizin- und Labortechnik GmbH, Dresden, Germany). Arbeitsanleitung Gesamt-Aluminium in Wasser/Abwasser, Brenzcatechinviolettmethode, 2004, based on APHA 3500-Al and ISO 10566
- Fe: Spectrophotometry,
  -   From 1996 until August 2005, flow-injection analysis with spectrophotometric detection: FIAstar (Tecator AB, Höganäs, Sweden). Application Note ASN 72-01/84 (Determination of iron in water by flow injection analysis (TPTZ method))
  -   Since September 2005, flow-injection analysis with spectrophotometric detection: FIAcompact (Medizin- und Labortechnik GmbH, Dresden, Germany). Arbeitsanleitung Gesamt-Eisen/Eisen (II) in Wasser/Abwasser, Labormethode für FIAcompact, Laboranleitung für FIAcompact, 2004, based on DIN 38406 Part 1, with ferrozine used instead of ortho-phenanthroline

- Si: Spectrophotometry,
  -   Before 1992, flow stream analysis^58^, conducted by Wasserwirtschaftsdirektion Havel, Potsdam, German Democratic Republic^48^
  -   1992–2009, flow-injection analysis with spectrophotometric detection: FIAstar (Tecator AB, Höganäs, Sweden). Application Short Note 4/92 (Determination of silica in water by flow injection analysis)
  -   Since 2009, flow-injection analysis with spectrophotometric detection: FIAstar 5000 Analyzer (Foss Analytical AB, Höganäs, Sweden). Application Note 5240 (Determination of silicate in water by FIAstar 5000) according to ISO 16264

- TIC and NPOC: catalytic oxidation, nondispersive infrared sensor (NDIR), quantification of CO_2_ released at high temperature from unfiltered water samples before and after acidification following the procedures described in the user manuals
  -   Between 1994 and October 2007, TOC-5000/5050 Analyzer, (Shimadzu, Kyoto, Japan)
  -   From November 2007 to November 2009, multi N/C 3100 Analyzer (Analytik Jena, Jena, Germany)
  -   Between December 2009 and 2018, TOC-VCPH Analyzer (Shimadzu, Kyoto, Japan)
  -   Between 2019 and 2020, TOC-LCPH Analyzer (Shimadzu Kyoto, Japan)

- DOC: catalytic oxidation, NDIR, TOC Analyzer (Shimadzu VCPH analyzer, Kyoto, Japan), determination of CO_2_ release from pre-filtered (0.45 µm pore size, filters as described above)) water samples, following the methods described in the user manual
- CaCO_3_: infrared gas analyzer (Infralyt 50 Saxon, Junkalor, Dessau, Germany)^59,60^, determination of CO_2_ released from filter residue after dry filtration (0.45 µm pore size, filters as described above) of water samples after dissolution in 10% HCl; CO_2_-C conversion to CaCO_3_ by applying a stoichiometric multiplication factor of 8.3
- Alkalinity: Titrimetry,
  -   Before 1991: Manual titrimetric analysis with methylorange or phenolphthalein^49^
  -   1991–2010: Titroprocessor 686 (Metrohm, Filderstadt, Germany), titration to pH 4.3, according to ISO 9963-1:1994
  -   Since 2011: Titrando 888 (Metrohm, Filderstadt, Germany), titration to pH 4.3, according to ISO 9963-1:1994

- Chl *a*: Spectrophotometry after overnight extraction in 90% acetone modified from references^61,62^ using membrane filters (ME 28, 47 mm 1.2 µm pore size, Whatman, Little Chalfont, United Kingdom). Before 1997, data were rounded to the nearest integer (µg L^−1^), thereafter to the nearest 0.1 µg L^−1^
  -   Until 2008, Lambda 2 spectrophotometer (Perkin Elmer, Waltham, MA, USA)
  -   Since 2009, U-2900 spectrophotometer (Hitachi, Tokio, Japan)

- Anions (Cl^−^, SO_4_^2−^) and cations (Na^+^, K^+^, Mg^2+^, Ca^2+^): Ion chromatography with conductivity detection following standardised protocols described in the user manual. Ion chromatographs used: Dionex DX-100 (2000–2006), Dionex 1000 (2007–2018), and Dionex Aquion (2019–2020) (all from Dionex Corp., Sunnyvale, CA, USA)

## Data Records

All data described here are available via the Freshwater Research and Environmental Database^63^ (FRED, https://fred.igb-berlin.de/) hosted by the IGB, Berlin, Germany. The dataset is split into three data packages: (1) Secchi depth and data of vertical profiles recorded with multiparameter probes, (2) water-chemical data determined spectrophotometrically or by other methods, and (3) anion and cation concentrations determined by ion chromatography. The three data packages have each been assigned a DOI and include metadata along with a data file in csv format. Over time the datasets and the corresponding metadata have been updated. This is visible from different DOI versions which are all visible and interlinked in FRED. Changes to previous versions are documented in the log file at the end of the metadata associated with each version. All files contain information on the lake, the study site, sampling date and sampling depth by which data between datasets can be linked.

1. Lake Stechlin vertical profiles of multiparameter probe measurements (1970–2020)^64^:
  T, O_2_, %O_2_, pH, conductivity, turbidity, chl *a*, PC, secchi
2. Lake Stechlin water chemistry data (spectrophotometry) (1970–2020)^65^:
  TP, SRP, TN, NO_2_^−^, NO_3_^−^, NH_4_^+^, Ca^2+^, Al, Fe, Si, NPOC, TIC, DOC, CaCO_3_, chl *a*, alkalinity
3. Lake Stechlin dissolved anions and cations (2000–2020)^66^:

Cl^−^, SO_4_^2−^, Na^+^, K^+^, Mg^2+^, Ca^2+^

## Technical Validation

Laboratory workflows followed standard protocols. Good laboratory practices were ensured throughout, including routine calibrations. Laboratory measurements were documented in laboratory note books and manually entered into a local database. Quality assurance of analytical procedures involved occasional participation in round-robin tests with multiple laboratories. The data were checked by an automated procedure to identify values beyond reasonable bounds, as well as by visual inspection. In cases of doubt, data entries were compared with the original records in laboratory note books. Discontinuities in trends were detected for three parameters (NPOC, TIC, spectrophotometrically determined Ca^2+^), the breakpoints coinciding with times when methods or instruments were changed. The data curation for these three parameters is described in detail in the following sections.

### Non-purgeable organic carbon (NPOC)

Non-purgeable organic carbon (NPOC) concentrations were measured on four different instruments. Five periods stood out where the measured NPOC concentrations deviated by more than 20% from the values expected based on the long-term trendline (Table 2), whereas similar deviations were not observed for any other variables. Furthermore, NPOC concentrations during these periods departed from the long-term trends also in two other clear-water lakes (Breiter Luzin and Lake Tiefwaren) and a brown-water lake (Große Fuchskuhle) included in IGB’s lake monitoring programme. These discrepancies suggest that measurement errors occurred during the five conspicuous periods.

**Table 2:** Summary of corrections applied to the raw data on non-purgeable organic carbon (NPOC) and total inorganic carbon (TIC) concentrations during different periods.

Parameter	Instrument	Period	Basis of correction	Correction factor
NPOC	Shimadzu TOC-5000/5050 Analyzer	April 2003 - Dec 2006	calibration curve	x 1.25
NPOC	Analytik Jena TOC Analyzer	Nov 2007 -July 2008	interpolation	x 0.8
NPOC	Analytik Jena TOC Analyzer	Dec 2008 - March 2009	interpolation	x 0.63
NPOC	Shimadzu TOC-VCPH Analyzer	Dec 2009 – Sep 2010	calibration curve	x 0.58
NPOC	Shimadzu TOC-VCPH Analyzer	May 2014- Sep 2014	interpolation	x 1.25
TIC	Shimadzu TOC-5000/5050 Analyzer	May 2006-Dec 2006	interpolation	x 1.33
TIC	Shimadzu TOC-VCPH Analyzer	Dec 2009-Dec 2010	calibration curve	x 0.86
TIC	Shimadzu TOC-VCPH Analyzer	April 2014 – Sep 2014	interpolation	x 0.74

For the two periods from April 2003 to December 2006 and from December 2009 to September 2010, slopes of the calibration curves differed by more than 20% from the slopes obtained both before and after these periods (Table 2). Consequently, we recalculated all concentrations based on the slopes of the calibration curves determined before and after the suspicious periods, resulting in 25% higher and 20% lower concentrations than originally measured during the first and second critical period, respectively.

**Fig. 3:**
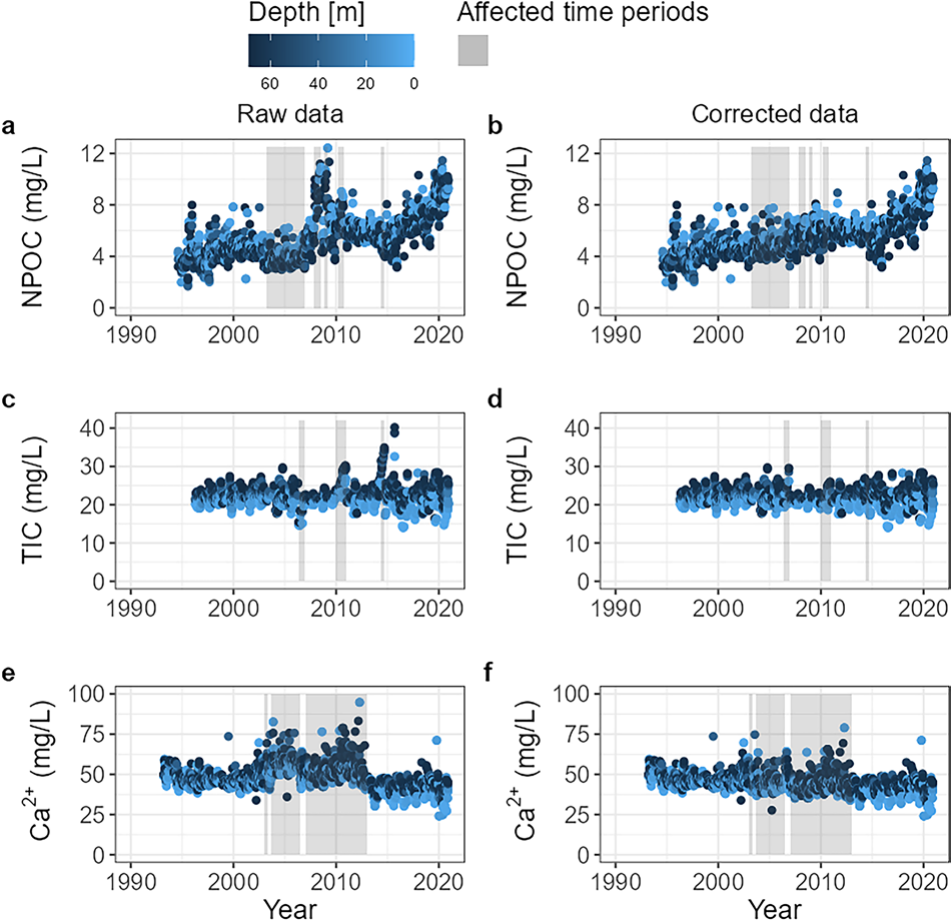
Time series of raw and corrected data for (**a,b**) non-purgeable organic carbon (NPOC), (**c,d**) total inorganic carbon (TIC), and (**e,f**) spectrophotometrically determined calcium concentrations determined in the main basin of Lake Stechlin, illustrating the effect of raw data corrections on temporal dynamics. Areas highlighted in grey indicate the affected time periods. Symbols of sampling values are coloured according to sampling depth as in Fig. 2.

In 2014, NPOC and TIC concentrations, which were measured on the same instruments, both deviated from the respective long-term trends, suggesting an instrument rather than a calibration issue. This explanation is also likely for the two periods from 2007–2008 and 2008–2009, because potential calibration errors could not be identified. To correct the NPOC data for these three periods, we first calculated an overall linear regression for the period from January 2004 onwards, ignoring all periods with identified issues as well as the last three years of the time series characterised by an increasingly steep rise in concentration (Fig. 3a–d). The linear regression yielded an R^2^ of 0.13 with a median value of 5.8 ± 1.0 mg/L. Next, we interpolated the missing values based on the linear regression and then calculated ratios of the median values of the originally measured concentrations (8.7, 9.1, 7.9 mg/L) and the median of the interpolation (5.5, 5.5, 6.0 mg/L) for each of the respective periods (2007–2008, 2008–2009, and 2014). Multiplication of these ratios with the originally measured concentrations resulted in corrected concentrations consistent with the long-term trend (median of respective time periods after correction: 5.5 ± 0.8, 5.5 ± 0.9, 6.3 ± 0.5), while maintaining the full variability of the original data (Table 2, Fig. 3a,b).

### Total inorganic carbon (TIC)

Total inorganic carbon (TIC) concentrations were determined with the same instruments used for the NPOC analyses. However, since different methods and calibration curves were used and calibrations were not always performed at the same time, periods with TIC calibration issues differed from those identified for NPOC (Table 2, Fig. 3c,d). An exception was in 2014 when the instrument issue affected both measurements. A calibration issue was identified for TIC samples taken between December 2009 and December 2010, when slopes differed by more than 20% from the slopes obtained both before and after this period. Consequently, concentrations for this period were recalculated based on a calibration curve established in February 2011, resulting in 40% lower concentrations than originally measured during the critical period. For the periods from May until December 2006 and from April until September 2014, we used the interpolation approach as described for NPOC, based on an overall linear regression calculated over the period from 2004 until 2017, with both suspicious periods excluded, resulting in a median of 21.5 ± 2.1 mg/L. We calculated correction factors (1.33 and 0.74) as the difference between the median values of the originally measured (16.4 and 29.1 mg/L) and the interpolated concentrations (21.7 and 21.5 mg/L), resulting in a corrected median of 21.8 ± 3.2 and 21.5 ± 2.6 mg/L for 2006 and 2014, respectively. In contrast to the NPOC data, no deviation from the general trend was observed for TIC concentrations during the operation of the Analytik Jena instrument between 2007 and 2009, probably because frequently encountered problems with the combustion tube or catalyst affected NPOC but not TIC measurements.

### Dissolved calcium

The spectrophotometric analyses of dissolved calcium (Ca^2+^) indicated increased concentrations between January 2003 and May 2003, from August 2003 to July 2006 and from January 2007 to December 2012. These elevated concentrations were not apparent in the ion chromatographic analyses. Specifically, the spectrophotometrically determined data exceeded those determined by ion chromatography by 30% between 2003 and 2007 and by 20% between 2007 and 2012. Consequently, the reported spectrophotometric data were corrected by applying a multiplication factor of 0.77, 0.77 and 0.83 for the three periods, respectively (Fig. 3e,f).

## Data Availability

All quality control steps and data corrections were performed using the software R. The corresponding R-code described in the section Technical Validation is available at the IGB GitHub repository (https://gitlab.igb-berlin.de/sabine.wollrab/fifty-years-monitoring-data-stechlin-cleaning/-/commit/19c78215b100203711693a4ec40f369008a63313). The corresponding raw data on which the data correction is based have been deposited in IGB’s data repository FRED^63^ (https://fred.igb-berlin.de/data/package/615), where they are linked to the respective cleaned data file via a DOI (10.18728/igb-fred-987.6).
